# The Evolution of Hand Proprioceptive and Motor Impairments in the Sub-Acute Phase After Stroke

**DOI:** 10.1177/15459683231207355

**Published:** 2023-11-13

**Authors:** Monika Zbytniewska-Mégret, Christian Salzmann, Christoph M. Kanzler, Thomas Hassa, Roger Gassert, Olivier Lambercy, Joachim Liepert

**Affiliations:** 1 Rehabilitation Engineering Laboratory, Department of Health Sciences and Technology, Institute of Robotics and Intelligent Systems, ETH Zurich, Zurich, Switzerland; 2 Kliniken Schmieder Allensbach, Allensbach, Germany; 3 Future Health Technologies, Singapore-ETH Centre, Campus for Research Excellence and Technological Enterprise (CREATE), Singapore, Singapore; 4 Lurija Institute for Rehabilitation Sciences and Health Research at the University of Konstanz, Konstanz, Germany

**Keywords:** stroke, recovery, proprioception, motor activity, hand, robotics

## Abstract

**Background:**

Hand proprioception is essential for fine movements and therefore many activities of daily living. Although frequently impaired after stroke, it is unclear how hand proprioception evolves in the sub-acute phase and whether it follows a similar pattern of changes as motor impairments.

**Objective:**

This work investigates whether there is a corresponding pattern of changes over time in hand proprioception and motor function as comprehensively quantified by a combination of robotic, clinical, and neurophysiological assessments.

**Methods:**

Finger proprioception (position sense) and motor function (force, velocity, range of motion) were evaluated using robotic assessments at baseline (<3 months after stroke) and up to 4 weeks later (discharge). Clinical assessments (among others, Box & Block Test [BBT]) as well as Somatosensory/Motor Evoked Potentials (SSEP/MEP) were additionally performed.

**Results:**

Complete datasets from 45 participants post-stroke were obtained. For 42% of all study participants proprioception and motor function had a dissociated pattern of changes (only 1 function considerably improved). This dissociation was either due to the absence of a measurable impairment in 1 modality at baseline, or due to a severe lesion of central somatosensory or motor tracts (absent SSEP/MEP). Better baseline BBT correlated with proprioceptive gains, while proprioceptive impairment at baseline did not correlate with change in BBT.

**Conclusions:**

Proprioception and motor function frequently followed a dissociated pattern of changes in sub-acute stroke. This highlights the importance of monitoring both functions, which could help to further personalize therapies.

## Introduction

Neurological injuries, such as stroke, lead to upper limb motor and/or somatosensory impairments.^1,2^ While more attention is paid to motor impairments in the clinical context, somatosensory impairments are in fact common after stroke and a prevalence of up to 64% has been reported.^3-5^ Somatosensory impairments have been shown to be associated with poor functional recovery and prolonged hospital stay.^4,6-8^ Among somatosensory modalities, proprioception, especially at the level of the hand, is of importance due to its relevance in the generation and control of dexterous movements.^9-11^ While it has been shown that motor recovery primarily occurs in the first 3 months post-stroke in the time-sensitive window of sub-acute stroke recovery,^12^ much less is known about the recovery of proprioception. In particular, it remains unclear how proprioception recovery relates to motor recovery, even though it could be essential in order to regain more complex motor function and fine hand control.^4,10,13^

A contributor to the limited knowledge on proprioceptive recovery is the challenge of quantifying it, as clinical assessments are not sensitive enough to detect subtle changes over time, suffer from ceiling/floor effects and are subjective.^14,15^ Robotic assessments may help overcome these challenges, as they rely on advanced sensing technology and can provide accurate stimuli during well-controlled tasks, such as finger displacement.^16-18^ For a better understanding of the underlying mechanisms and for an objective assessment of central nervous system damage, as well as for recovery prediction, neurophysiological assessments can be used (somatosensory or motor evoked potentials SSEP/MEP to inform on the integrity of central somatosensory or motor tracts).^19-21^ As such, a combination of behavioral and neurophysiological measures of proprioception and motor function in a comprehensive, longitudinal study is promising to bring new perspectives on the topic of stroke recovery.

Previous studies, using clinical behavioral measures, have shown that improvement over time is expected for both somatosensory and motor function of the upper limb, given the general state of heightened neuroplasticity in the sub-acute phase after stroke.^4^ A considerable inter-individual variability has been reported in the magnitude of motor recovery, with some individuals showing very poor recovery, often explained by the lesion affecting the corticospinal tract and other stroke-related or personal factors.^22-24^ It is, however, unclear whether a similar pattern of variability could be expected for proprioception. Motor and somatosensory function have been shown to be longitudinally associated early after stroke,^4,13^ but their recovery might follow different time courses.^25^ For example, it has been suggested that severe initial somatosensory impairment does not directly compromise motor recovery.^4^ Moreover, the magnitude and timing of motor and proprioceptive recovery has been shown to be dissociated for some patients, as measured by a sensitive robotic assessment for the proximal joints of the upper limb.^25^ It is not yet understood what possible reasons for this dissociation are and whether a similar pattern of changes could be observed at the level of distal joints of the upper limb, where the importance of the interplay between motor function and proprioception in the execution of functional tasks is paramount.^11,26^ In fact, it has been proposed that the recovery of somatosensory function might be a prerequisite to reach full score on the Fugl–Meyer Upper Limb Motor Assessment (FMA), which involves being able to perform dexterous hand movements, such as pinching.^4,27^ Impaired proprioception has also been shown to affect fine motor skills and the ability to improve in the Box and Block test.^28^

In this observational study, we aim to quantify how proprioception and motor function of the hand evolve over time in the sub-acute phase after stroke using sensitive, previously validated robotic assessments of the index finger metacarpophalangeal joint.^17,29^ We hypothesized that (i) different patterns of proprioceptive and motor impairment and recovery will be observed among sub-acute stroke subjects as measured by the robotic assessments, and that (ii) these patterns can be explained by specific demographic, stroke-related, behavioral, and neurophysiological factors, and that (iii) good functional recovery of the hand relies on both proprioception and motor function.

## Methods

### Participants

Participants with stroke were recruited as soon as they entered the in-patient rehabilitation clinic (Kliniken Schmieder, Allensbach, Germany). During their stay at the clinic, participants received standard, personalized, neurorehabilitation, which can be considered usual care. Inclusion criteria for the study were: age >18 years, diagnosis of stroke (ischemic or hemorrhagic), less than 3 months post-stroke, and the ability to passively move the metacarpophalangeal (MCP) joint by at least 20°. Exclusion criteria were: inability to understand instructions, pain when moving the MCP joint, diagnosis of visuospatial neglect (Bells Test^30^) or aphasia. All participants gave written informed consent before participating in the study. The study was approved by the Ethics Commission of Baden-Württemberg F-2016-126 and registered as a clinical trial.^a^

### Study Protocol

At study inclusion (baseline, T1), demographic information (age, gender, handedness evaluated with the Edinburgh Handedness Inventory, more affected side, date, and type of stroke) was collected and robotic (primary outcome measures), clinical and neurophysiology assessments (secondary outcome measures) were performed. Each group of assessments was performed in a separate 30 minutes to 1 hour session. After 4 weeks (discharge, T2), robotic and clinical assessments were repeated, unless discharge from the clinic occurred earlier, in which case the measurement was performed at the time of discharge (at least 2 weeks after inclusion). Only data from the most affected side were considered for analysis.

### Robotic Assessments

*Apparatus*: ETH MIKE (Motor Impairment and Kinaesthetic Evaluation) is a one degree-of-freedom end-effector robot, which can provide accurate displacement to the index finger (MCP joint) and measure its resulting response (position, force, velocity).^17,31^ During an experiment, participants are seated in front of the device, grasping a handle, with their index finger stretched and attached to the end-effector with Velcro straps. A tablet computer is placed directly above the hand, serving as a visual display during the assessments and blocking vision of the participant on the hand.

In this study, 4 different, previously validated assessment tasks were performed.^17^ One task assesses proprioception (position sense) and does not require active movement of the tested finger. Three other robotic tasks comprehensively assess distinct subcomponents of hand motor function (force, range of motion, velocity). All tasks rely on paradigms commonly used in the literature and are motivated through physiological mechanisms of proprioception and motor function.^2,32^

*Gauge Position Matching*: in this task assessing proprioception, the index finger is passively displaced by the robot, and the perceived finger position needs to be indicated by pointing on the tablet screen above the hand^17,33^ (11 trials, randomized positions within a range 10-30° from starting position). The outcome measure (also referred to as task metric) is the Absolute Error (AE) between actual and indicated positions (in °), which has shown excellent test-retest reliability in previous work with people after stroke (Intraclass Correlation Coefficient ICC(A,k) = 0.90).^17^

*Maximum Fingertip Force in Flexion*: in this task, the end-effector is fixed, and the participant needs to press as strong as possible in flexion direction (3 trials). The maximum force is measured using a force sensor located at the end-effector. The outcome measure (ie, task metric) is the mean maximum force over the 3 trials (denoted as Flexion Force FF, in N). This metric has shown excellent test-retest reliability, as reported in our previous work (ICC(A,k) = 0.97).^17^

*Active Range of Motion*: in this task, the participant moves the finger to the maximum position in flexion and then in extension (3 trials). The outcome measure is the mean active range of motion across the 3 task repetitions (Active Range of Motion AROM, in °) and the ICC(A,k) has been reported as 0.97.^17^

*Maximum Velocity in Extension*: after participants finger is passively moved to a starting position in flexion, he/she needs to move as fast as possible to a position in extension direction, corresponding to a target position displayed on the target screen (5 trials). The outcome measure is the mean of 3 maximum velocity values across the 5 trials performed (Extension Velocity, EV, in °/s, ICC(A,k) = 0.98)^17^).

For more details on the apparatus, assessment tasks, as well as selection and validation of outcome measures, please refer to Supplemental Material, Figure SM1 and previous work.^17,29^

### Clinical Assessments

The following clinical assessments were performed: FMA^27^ for the general assessment of upper limb motor function (scores: 0-66), kinaesthetic Up-Down test as a part of Nottingham Sensory Assessment (kUDT)^34^ for the assessment of finger proprioception (score: 0-3), Box & Block Test (BBT)^35^ for the assessment of functional hand use (unit: blocks/min), Montreal Cognitive Assessment (MoCA)^36^ for the assessment of cognitive function (score: 0-30).

### Neurophysiology

A standard protocol was used to obtain SSEP and MEP.^20^ In brief, participants were first seated in a comfortable chair. To obtain SSEP, the median nerve was electrically stimulated at the level of the wrist, and recordings were taken from C3′ and C4′, respectively, using silver chloride skin electrodes. The reference electrode was placed over Fz. The latency and amplitude of the N20 potential were analyzed. To obtain MEP, motor cortex was stimulated using a circular coil connected to a Magstim device (Whitland, United Kingdom). Recordings were obtained from the first dorsal interosseous muscle using surface electrodes. MEP amplitude and latency were considered. The SSEP/MEP response of the affected side was categorized as abnormal (impaired) if amplitude was less than 50% of the amplitude obtained from the unaffected side or if latency was more than >1.2 milliseconds longer than in the unaffected side.^20^ It was categorized as absent if no response was obtained or if the response had no identifiable N20 peak.

### Statistical Analysis

Descriptive statistics are reported as mean and standard deviation (SD). Paired sample *t*-tests were used to evaluate statistically significant differences between T1 and T2 at the population level. Further, to specifically investigate individual changes, participants were categorized depending on whether a considerable improvement in proprioception and/or motor function, as measured by the robotic outcome measures, was observed. This was to ensure the investigated improvement could be attributed to recovery rather than measurement noise. For proprioception, considerable improvement was defined as change between T1 and T2 larger than the smallest real difference (SRD) of AE or if the participant changed from impaired to non-impaired (defined as AE above age-matched control mean + 2 × SD^18^) within the measured timeframe. For motor function, considerable improvement was defined as change above the SRD or change from impaired to non-impaired in 1 of 3 motor outcome measures (FF, AROM, or EV). The SRDs and impairment thresholds for robotic measures were derived based on our previous work on outcome measures validation^17^ and for clinical measures based on literature,^36,37-40^ as reported in Table SM1. Accordingly, it was possible to divide the participants into those with “corresponding patterns of change” (when both proprioception and motor function improved/did not improve) or “dissociated patterns of change” (when only one of the functions improved). This analysis was further refined by conducting a linear mixed effect model analysis, where the change in proprioception between T1 and T2 (∆AE) was chosen as a dependent variable and changes in the subcomponents of motor function were considered as fixed factors (∆FF, ∆AROM, ∆EV). Further, the groups that did and did not considerably improve according to the robotic measures were compared in terms of personal, behavioral, and neurophysiological factors using Kruskal–Wallis one-way analysis of variance. The following baseline characteristics were considered: proprioceptive and motor impairment (measured by robotic assessments), MEP and SSEP category (normal, impaired, or absent), time since stroke, age, gender, cognitive function (MoCA), type of stroke (ischemic or hemorrhagic), lateralization (left or right hemispheric stroke). To further investigate the factors influencing change, linear mixed effect models were built with change in proprioception and change in motor function as dependent variables and the following variables as fixed factors at baseline: kUDT, FMA, time since stroke, age, gender, MoCA, stroke type, lateralization. This analysis was done considering all participants. Finally, to analyze the potential relationship between hand impairments and the functional hand use (BBT), Pearson (*r)* or Spearman’s rank (ρ) correlation was used, considering sample size requirements for the choice of the correlation type (Pearson was used for sample sizes larger than 30).^41^

## Results

### Participants

Fifty participants were included in the study, 5 dropped out (had only 1 measurement hence not suitable for the longitudinal analysis). Forty-five participants (aged 67.82 ± 10.52 years, 16 females, 43 right-handed, 20 left hemispheric stroke, 34.38 ± 15.12 days since stroke at T1, 34 ischemic stroke) that completed the robotic assessments at 2 measurement timepoints were included in the analysis. For 38 individuals the time difference between baseline and discharge was 4 weeks, while 7 participants were discharged from the clinic earlier (discharge assessments after 2 weeks from baseline), but both groups are considered together in the analysis (Table 1), as no significant difference was found in the amount of change between these groups. Out of the 45, 43 had all baseline and discharge clinical assessments completed and their results are presented in Tables 1 and 2. Moreover, SSEP was collected from 28 participants at T1. MEP data was available for 38 individuals. The reasons for dropouts or missing measurements were patient’s illness, earlier discharge, unwillingness to further participate in the study or staff unavailability. Details on the total number of participants screened, included, and analyzed for this study are available in the PRISMA diagram in the Supplemental Material (Figure SM16).

**Table 1.** Group Results of Robotic and Clinical Assessments at Baseline and Discharge.

Assessment	Category	Baseline (*T*1)	Discharge (*T*2)	*t*-Test *P*	%∆ consid.
AE (°)	Proprio.	13.26 ± 5.65	11.76 ± 6.40	.041	22% (10/45)
kUDT [0-3]		1.95 ± 1.19	2.21 ± 1.06	.003	21% (9/43)
FF (N)	Motor	13.39 ± 13.13	15.77 ± 12.46	.003	31% (14/45)
AROM (°)		51.55 ± 25.38	57.74 ± 24.44	.005	22% (10/45)
EV (°/second)		176.53 ± 174.58	189.74 ± 173.36	.317	33% (15/45)
FMA [0-66]		32.26 ± 23.75	38.36 ± 22.72	<.001	51% (22/43)
BBT (#/minute)	Functional	19.21 ± 20.00	25.37 ± 23.20	<.001	44% (19/43)
MoCA [0-30]	Cognitive	21.95 ± 4.94	23.21 ± 4.41	.004	33% (14/43)

Abbreviations: Proprio., Proprioception; AE, Absolute Error; kUDT, kinesthetic Up Down Test; FF, Flexion Force; AROM, Active Range of Motion; EV, Extension Velocity; FMA, Fugl-Meyer Upper Limb Motor Assessment; BBT, Box & Block Test; MoCA, Montreal Cognitive Assessment; %∆ consid., % of subjects improving considerably according to our established criteria.

For robotic assessments (AE, FF, AROM, EV) N = 45, for clinical (kUDT, FMA, BBT, MoCA) N = 43. Detailed results for each participant are shown in Table 2.

**Table 2.** Clinical, Robotic, and Neurophysiology Measures for Each Participant.

*T*1												*T*2									
#	kUDT T1	FMA T1	BBT T1	MoCA T1	AE (°) T1	FF (N) T1	AROM (°) T1	EV (°/second) T1	SSEP T1	MEP T1	TSS (days)	Change group	kUDT T2	FMA T2	BBT T2	MoCA T2	AE (°) T2	FF (N) T2	AROM (°) T2	EV (°/second) T2	Early discharge
1	0	4	0	22	13.04	2.59	45.24	32.36	Absent	Impaired	43	None	0	9	0	22	14.59	6.70	54.86	8.39	0
2	3	4	0	17	14.99	0.66	3.87	52.47	Absent	Impaired	40	None	3	4	0	23	17.13	0.44	6.39	25.95	0
3	0	56	19	24	19.52	11.20	75.66	293.28	Absent	Normal	30	None	2	63	37	26	11.05	15.55	75.91	276.16	0
4	0	4	0	26	19.27	3.07	45.43	8.02	Absent	Impaired	24	None	0	10	0	24	19.31	5.99	60.00	22.35	0
5	0	10	0	26	15.72	5.49	48.40	9.69	NaN	NaN	41	None	1	21	1	30	22.05	7.63	50.22	20.72	0
6	1	9	0	25	16.92	4.18	50.41	17.12	Absent	Impaired	48	None	1	19	0	25	20.53	7.41	55.10	43.06	0
7	2	4	0	25	22.48	1.66	28.78	4.91	Normal	Absent	65	None	2	4	0	23	20.36	1.63	22.98	5.77	0
8	0	4	0	29	20.00	0.73	0.99	5.02	Impaired	Impaired	80	None	0	9	0	26	20.00	1.51	15.64	3.89	0
9	3	18	7	24	7.42	5.48	44.59	78.10	Normal	Impaired	55	None	3	24	8	26	5.48	9.03	54.59	86.55	0
10	2	0	0	19	20.34	1.46	23.09	7.94	Impaired	Absent	45	None	2	7	0	18	18.24	0.72	2.60	6.78	0
11	3	23	4	25	16.94	3.15	58.66	67.73	Impaired	Normal	27	None	3	35	19	22	14.43	3.98	60.00	71.98	1
12	3	4	0	24	8.03	0.38	0.13	32.64	Normal	Absent	23	None	3	5	0	25	4.53	0.38	0.13	32.64	1
13	1	19	0	9	16.51	19.58	79.26	515.03	Normal	NaN	31	None	NaN	NaN	NaN	NaN	22.18	19.40	59.52	134.47	0
14	2	51	31	3	14.40	5.89	60.00	77.14	Impaired	Normal	64	None	3	54	15	6	10.82	7.14	52.26	53.58	1
15	1	4	0	26	15.32	1.30	58.35	3.95	NaN	Impaired	36	None	2	7	0	27	19.85	3.47	60.00	3.96	0
16	3	37	30	23	7.21	49.55	59.95	230.36	Impaired	Impaired	25	None	3	49	37	22	15.22	41.00	59.97	238.14	0
17	3	63	46	25	7.81	37.91	66.94	352.52	Normal	Normal	22	None	3	65	50	23	6.05	30.74	80.87	297.87	1
18	3	59	33	19	10.13	28.25	70.75	265.52	NaN	Impaired	24	None	3	65	41	17	6.51	31.85	65.70	227.90	0
19	3	49	44	25	17.74	16.18	59.99	307.51	Impaired	Normal	25	Both	3	56	56	23	10.63	10.76	66.52	308.45	0
20	3	61	52	25	13.76	14.32	68.69	433.76	Impaired	Impaired	43	Both	3	65	48	26	9.92	28.85	87.82	520.71	0
21	3	50	48	24	13.65	24.73	59.99	212.07	Impaired	Impaired	29	Both	3	61	60	27	3.84	33.54	71.48	307.69	0
22	3	59	29	21	13.60	13.21	69.62	536.39	Normal	Normal	19	Both	3	62	42	24	9.08	23.22	80.01	408.14	0
23	3	14	0	19	11.62	2.30	32.62	16.94	Normal	Impaired	31	Both	3	26	8	19	8.74	3.33	50.34	5.13	0
24	3	49	30	28	10.89	22.11	66.67	168.93	Normal	Normal	16	Both	3	60	63	25	4.82	32.84	59.97	254.74	0
25	3	57	47	17	20.85	22.80	82.51	196.30	NaN	Impaired	62	Both	3	60	51	18	11.19	17.54	63.24	328.91	0
26	2	37	17	19	15.44	14.82	65.04	272.12	NaN	Normal	48	Both	2	44	26	23	9.50	20.25	59.99	240.94	0
27	3	58	58	22	3.12	38.45	80.05	525.32	Normal	Impaired	31	Motor	3	58	58	26	4.90	36.46	101.02	502.98	1
28	3	61	57	20	6.57	31.26	73.60	440.40	Normal	Normal	31	Motor	3	62	59	22	5.07	38.02	85.37	349.54	0
29	1	37	13	12	24.99	5.36	52.79	4.45	Impaired	Impaired	22	Motor	2	48	32	21	24.26	15.67	59.99	131.11	0
30	0	4	0	20	19.53	0.98	9.32	49.14	Absent	NaN	39	Motor	0	21	0	23	17.53	14.37	59.79	38.64	0
31	0	56	26	28	6.09	30.82	83.17	421.76	Absent	Normal	18	Motor	0	63	39	29	4.70	26.19	88.04	495.15	0
32	0	4	0	24	18.24	1.22	20.32	4.17	Absent	Absent	22	Motor	0	7	0	23	19.40	2.34	47.94	4.78	0
33	3	53	22	20	16.07	14.50	59.99	247.74	Absent	Normal	39	Motor	3	54	43	21	13.02	12.28	70.92	348.89	0
34	0	8	0	20	9.38	0.86	21.57	11.97	Absent	Impaired	21	Motor	2	33	0	21	13.19	6.81	59.35	82.84	0
35	3	48	44	25	9.71	15.48	60.01	108.09	Normal	NaN	27	Motor	3	44	55	28	5.28	20.57	60.00	172.80	0
36	2	64	42	26	4.87	34.98	81.73	232.92	Normal	NaN	42	Motor	NaN	NaN	NaN	NaN	3.57	40.50	79.87	305.08	1
37	2	37	21	24	21.12	3.95	59.95	86.38	Absent	Impaired	61	Motor	2	44	34	26	18.38	10.40	60.02	265.93	0
38	2	19	0	23	6.67	9.04	49.67	5.38	NaN	Absent	28	Motor	3	24	0	27	7.47	11.36	59.37	4.96	0
39	3	66	43	23	9.63	38.84	95.30	565.24	NaN	NaN	21	Motor	3	66	54	27	4.67	34.34	107.10	642.91	0
40	2	35	9	9	7.47	11.90	28.90	99.60	Normal	Absent	28	Motor	2	45	29	11	4.84	11.16	42.56	236.32	0
41	2	8	0	21	6.72	0.45	1.25	52.85	NaN	Impaired	16	Motor	2	20	0	23	5.08	5.19	34.00	32.31	0
42	1	63	45	22	6.76	16.80	63.78	386.20	NaN	Impaired	24	Motor	2	64	51	23	20.58	20.38	60.01	451.04	1
43	2	30	11	20	3.37	10.34	60.00	157.98	NaN	Normal	24	Motor	2	41	36	27	5.09	12.36	62.98	262.63	0
44	2	4	0	24	14.42	0.30	11.77	90.06	Impaired	Absent	15	Sensory	3	4	0	24	7.35	0.57	0.80	15.65	0
45	3	66	40	27	18.32	23.94	80.97	256.37	Impaired	Normal	42	Sensory	3	66	39	26	8.77	25.86	83.11	259.83	0

Abbreviations: kUDT, kinestheatic Up-Down Test; FMA, Fugl-Meyer Upper Limb Motor Assessment; BBT, Box & Block Test; MoCA, Montreal Cognitive Assessment; AE, Absolute Error; FF, Force Flexion; AROM, Active Range of Motion; EV, Extension Velocity; SSEP, Somatosensory Evoked Potentials; MEP, Motor Evoked Potentials; TSS, Time Since Stroke; Change group, category of considerable improvement either in both proprioception (sensory function) and motor function, in only proprioception, only in motor function or in none of the functions; Early discharge, if *T*2 assessment performed earlier than after 4 weeks.

### Relationship Between Changes in Proprioception and Motor Function

**Figure 1.**
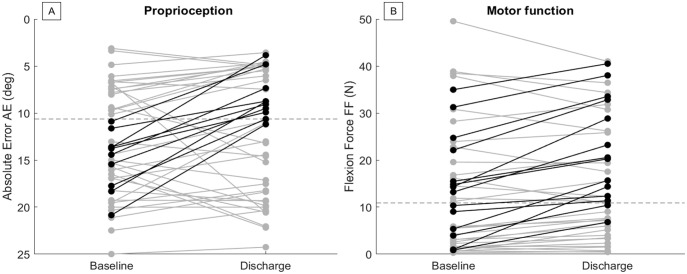
Changes over time of proprioception and motor function, measured by 2 of the robotic task metrics (Absolute Error (AE) in (A) and Flexion Force (FF), 1 of 3 motor metrics, in (B). In black are marked individuals that improved considerably = change > smallest real difference (SRD) or change from impaired to non-impaired. The dashed lines mark impairment thresholds (based on mean + 2SD of neurologically intact age-matched controls). There is a large variability in individual changes. For each metric there are 10, respectively 14, participants that considerably improved (black), but for the majority of the participants changes were too small to be classified as considerable (grey). While the trend of increasing performance over time was prominent, there were also few individuals that considerably decreased in proprioception or motor function. Namely, 3 participants considerably decreased in proprioception and 3 in motor function. Higher FF and smaller AE indicate better performance. The y-axis of Figure 1(A) was therefore flipped to enhance comparability between Figures (A) and (B).

At the population level, significant improvements were seen in both proprioception and motor function over the course of the study, as measured by the robotic assessments (*t*-test *P*-value < .05 for all but EV). The average change was smaller than SRD and on average participants remained impaired at discharge according to all outcome measures but FF (Table 1). Visualization of the longitudinal changes in robotic outcome measures can be found in Figure 1 and Figure SM2.

Based on the robotic assessments, 4 groups with distinct patterns of change were identified, namely considerable improvement in: both proprioception and motor function (8/45 individuals), motor function only (17/45), proprioception only (2/45), neither proprioception nor motor function (18/45). Overall, 19/45 (42%) of all study participants had a dissociated pattern of change in proprioception and motor function (only one of these functions considerably improved). The finding that dissociated patterns of change in proprioception and motor function was frequent was confirmed by the results of the linear mixed effect model, as ∆AE was not related with ∆FF, ∆AROM or ∆EV (*P* = .339, .302, and .230, respectively, N = 45, see Figure SM3 for details). Within the group with motor change only, 12/17 participants had non-impaired proprioception at baseline. Within the group with proprioception change only, 1 of 2 participants had non-impaired motor function at T1. Considerable improvement in motor function was still possible with motor function initially not categorized as impaired (6 participants), while it could not be observed for proprioception (ie, participants already classified as non-impaired in proprioception at T1 could not further improve).

**Figure 2.**
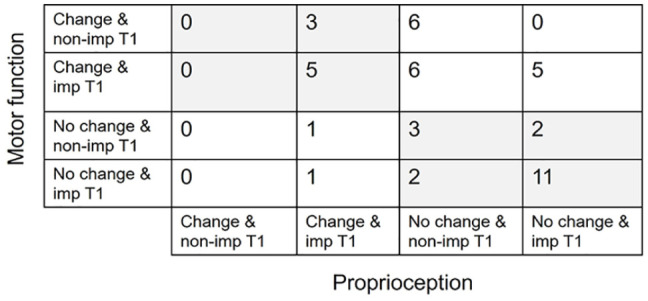
Matrix showing how changes in proprioception and motor function (quantified using the robotic outcome measures) were related with each other, also already including information whether a given modality was impaired at baseline (T1), for all participants (N = 45). The fields in grey indicate corresponding patterns of changes while white fields show dissociated patterns of changes. Abbreviations: change, considerable improvement; non-imp., non-impaired; imp., impaired.

It is therefore important to additionally analyze the subgroup of participants with impaired proprioception at baseline. Among this group, 75% (21/28) of participants showed a corresponding pattern of change (8 considerably improved in both modalities, 13 improved in neither). For 25% (7/28) of participants, change was dissociated, 2 subjects considerably improved in proprioception only, while 5 subjects improved in motor function only (Figure 2). The linear mixed effect model analysis still revealed a dissociated pattern of change for this subgroup (*P* = .615, .276, and .053 for ∆FF, ∆AROM, and ∆EV respectively, N = 28, Figure SM4).

### Possible Explanations for the Observed Patterns of Changes

*Behavioral Factors*: Participants that did not considerably improve in motor function, as measured by the robotic assessments, had on average lower baseline motor function than subjects that did improve (FF: 4.46 ± 5.05N (N = 15) vs 15.83N ± 12.03N (N = 25), *P* = .003, similar results for the other motor metrics, Figure SM9A). There was no significant difference in baseline proprioception between the group that did and did not improve in proprioception (AE: 15.03 ± 3.11° (N = 10) vs 16.06 ± 4.27 (N = 15), *P* = .318, Figure SM9B).

**Figure 3.**
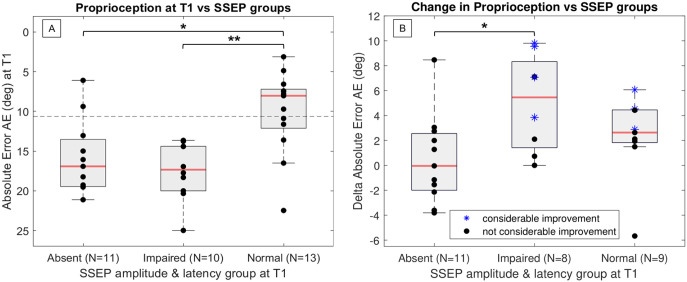
Grouping of participants based on SSEP scores to compare Gauge Position Matching AE at T1 (A) and delta AE (B) between the absent, impaired, and normal SSEP response groups. In (A) the dashed line indicates impairment threshold. Larger delta AE more improvement, while smaller AE indicates better performance, hence the y-axis of Figure 3(A) was flipped to enhance readability. The reason for “Impaired” and “Normal” groups having missing data for (B) is due to the missing discharge measurement for these patients (no delta AE available).

*Neurophysiological Factors*: on average participants with absent SSEP at T1 had impaired proprioception at T1 and did not change in proprioception (AE at T1 = 15.83° ± 4.68° > 10.63°, ∆ = 0.48 ± 3.57°; N = 11, Figure 3). Correspondingly, on average participants with absent MEP at T1 had impaired motor function at baseline and did not change in any subcomponents of motor function as measured by the robotic assessments (FF at T1 = 3.71 ± 4.72N < 10.93N, ∆ = 0.95 ± 1.55N, N = 7, Figure SM10, similar results for other motor metrics are shown in Figure SM10).

Now, using neurophysiological results to further understand the dissociation between motor and proprioceptive changes, 4/5 participants that considerably improved in motor function, but not in proprioception (considering only those with impaired proprioception at T1), had preserved MEP (ie, detectable response, either impaired or normal), but absent SSEP (ie, no response detected). Correspondingly, 1 participant that considerably improved in proprioception, but not in motor function despite improvement potential (impaired at T1), had preserved SSEP, but absent MEP. Conversely, participants with considerable improvement in both motor function and proprioception had preserved MEP and SSEP (8/8 preserved MEP, 6/8 preserved SSEP, for 2 missing SSEP data, visualized in Figure 3 and Figure SM10).

*Demographic and Stroke-Related Factors*: There was no statistically significant difference in age, gender, type of stroke, stroke side (left or right hemispheric stroke), or cognitive function between the group that considerably improved in at least 1 domain and the group that did not change (Figure SM11). There was a significant difference in the time since stroke between the groups (30.52 ± 12.73 days vs 43.47 ± 16.60 days, *P* = .008). However, the results of the linear mixed effect model indicated that none of the considered factors were significantly affecting change in proprioception (∆AE), nor in motor function (∆FF, ∆AROM, ∆EV), as detailed in Figures SM5 to SM8.

### Relationship Between Changes in Hand Impairments and Functional Hand Use

**Figure 4.**
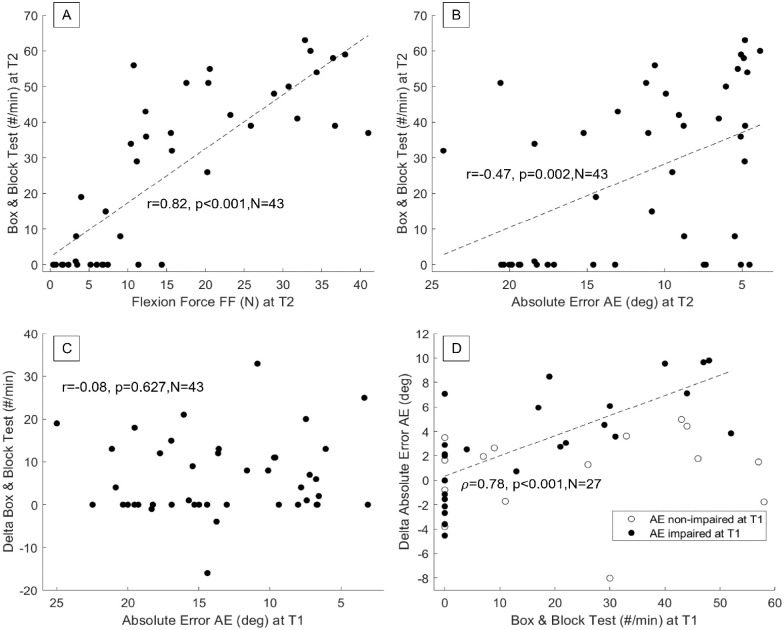
There is a relationship between hand impairments and functional hand use at discharge. Both motor function (here Force Flexion, (A)) and proprioception (Absolute Error, (B)) correlated with the skilled hand use at discharge, measured by Box & Block Test (BBT). (C) Impaired proprioception at baseline did not correlate with functional recovery, as measured by delta BBT. (D) At least partially preserved hand function was needed for improvement in proprioception, especially considering individuals with improvement capacity (ie, impaired at T1).

Participants that performed best in BBT at discharge had higher maximum fingertip force (Figure 4A) and smaller proprioceptive error at the same time point (Figure 4B). There was a strong significant correlation between BBT and Force Flexion at T2 (Pearson *r* = 0.82, *P* < .001, N = 43, comparable result for other motor measures AROM and EV, Figure SM8), and a moderate significant negative correlation between BBT and AE at T2 (*r* = −0.47, *P* = .002, N = 43). Further, there was no significant correlation between AE at T1 and change in BBT between baseline and discharge (*r* = −0.08, *P* = .63, N = 43, Figure 4C). Finally, when considering only individuals with capacity to improve (ie, impaired at T1), there was a strong significant correlation between BBT at T1 and change in AE (Spearman ρ = 0.78, *P* < .001, N = 16, Figure 4D), indicating that the higher the performance in the BBT at baseline, the larger the improvements in proprioception. The correlations remained significant when the values in floor of the BBT scale were removed (Figure SM12).

## Discussion

In this work we longitudinally assessed hand proprioception and motor function over up to 4 weeks in 45 sub-acute stroke participants undergoing inpatient rehabilitation (usual care). A dissociated pattern of change of proprioception and motor function was frequently observed. We showed that the lack of considerable improvement in 1 function was linked to a severe lesion of central somatosensory or motor tracts (absent SSEP/MEP) or a well-preserved function at baseline. We also found that functional hand use at baseline correlated with proprioceptive improvement, suggesting that active hand use is important for proprioceptive recovery. Recovered or intact proprioception is in turn needed to reach better functional performance at discharge, highlighting the intertwined relationship between proprioception and motor function.^42,43^

### Dissociated Pattern of Proprioceptive and Motor Changes Was Frequent

Although the majority of patients showed corresponding patterns of change, a substantial part (25-42%, depending on whether only the subgroup with measurable impairment in proprioception or all participants are being considered) showed dissociated recovery patterns. Moreover, the linear mixed effect models revealed a lack of relationship between change in proprioception and change in motor function. Our finding that dissociated changes frequently occurred is supported by recent work, which used another technology-based assessment tool of proximal joints of the upper limb, that also pointed to dissociated recovery of proprioception and motor function for several sub-acute stroke participants (32% of subjects had inconsistent performance when comparing motor and proprioceptive recovery).^25^ It underlines the importance of carefully studying these impairments and their interaction, which is hardly possible with only conventional clinical measures. Indeed, using robotics it becomes possible to detect changes previously not captured by clinical measures. For example, in this study we found that among 10 individuals that considerably improved in the robotic Position Matching task, for 8 of them this change was not detected by the clinical assessment (kUDT) due to the ceiling effect of the scale (Table SM2). Understanding of these different recovery profiles, now becoming possible thanks to sensitive measurement tools, could in turn bring a new perspective on treatment requirements. For instance, if identified that over time improvement predominantly occurs in 1 modality, it could be recommended to adjust the therapy plan to further integrate the other modality, or accordingly revise therapy objectives.

### Factors Explaining the Dissociation Include Baseline Impairment Severity and Absent Neurophysiological Response

We found 2 possible explanations for the dissociation of proprioceptive and motor changes observed in some participants. Firstly, the lack of considerable change in 1 modality could be explained by an absence of impairment in that modality at baseline. Indeed, it has been shown previously that proprioceptive and motor impairments may occur independently for many individuals with sub-acute stroke (impairment in only 1 modality in 38% of subjects).^44^ In our study, it was especially the case for proprioception, as we found that 12 of the 17 individuals that improved in motor function only had well-preserved proprioception at baseline. Participants with proprioceptive function within healthy norm at baseline could not change enough to reach the threshold of “considerable improvement,” due to the properties of the scale and healthy norms being generally elevated for older adults.^45-48^ On the contrary, well-preserved motor function at baseline was uncommon (13% of participants).

Secondly, dissociation between the changes in proprioception and motor function could be linked to different impairment severity of the respective neural pathways. Poor motor recovery has often been linked to severe damage to the corticospinal tract, detectable by absent MEP response.^22,49,50^ While MEP measures are already established as biomarkers of motor recovery,^19^ SSEP responses are less commonly used.^19^ In this study we found that individuals with absent SSEP improved the least in proprioception, while all individuals that considerably improved in proprioception had preserved SSEP, indicating an explanatory value of SSEP with respect to proprioceptive improvement, which adds to existing research linking SSEP with functional recovery.^21^ Interestingly, individuals with considerable improvement only in motor function and with impaired proprioception at baseline had absent SSEP, but preserved MEP response at baseline. The same was true for 1 individual who improved in proprioception only, in this case MEP was absent, but SSEP present at baseline. Although sensory and motor tracts are located nearby and are to a large extent overlapping in the brain,^28,51^ it is possible, depending on the location and size of the lesion due to stroke, that only one of those tracts is predominantly affected, explaining that dissociation, as has been reported for exclusively motor strokes.^52^ Indeed, other work on the comparison of MEPs and SSEPs has revealed that each may be affected independently by stroke, confirming that they rely on anatomically discrete pathways.^21,53^

### Proprioceptive Impairment Did Not Prevent Functional Improvement, But Ability to Use the Hand Functionally at Baseline Related With Proprioceptive Improvement

In contrary to our initial hypothesis, impaired position sense at baseline did not prevent improvement in functional hand use. Specifically, the baseline AE was not correlated with the change in BBT. This finding is in line with previous research, which showed that severe somatosensory impairment does not directly compromise motor recovery in the sub-acute phase after stroke.^4,25^ However, it partly contradicts other work in chronic stroke that linked baseline proprioceptive impairment to the lack of treatment gains in the BBT.^28^ In that study a specific intervention stimulating proprioceptive feedback was provided, since modulation of proprioceptive integration has been shown to influence motor learning.^54^ In chronic stroke the process of motor skill recovery is mostly driven by therapeutic interventions, in this case influenced by the ability to integrate the additional proprioceptive feedback provided.^28^ In the sub-acute phase, when spontaneous recovery can be expected, it might rather be the integrity of corticospinal tract that drives changes in BBT, as it does for basic motor function and as indicated by individuals with the largest improvement in BBT having normal MEP responses (Figure SM9).

Other regions than the corticospinal tract are also likely involved in recovery of manual dexterity, such as regions responsible for sensorimotor integration (eg, Posterior Parietal Cortex).^55,56^ Linked to that, we showed that, as hypothesized, both proprioception and motor function were needed to achieve best performance in BBT at discharge. Hence, recovery of these functions, or the way they are preserved at baseline, as well as the ability to integrate them, were needed for reaching fine motor skill at discharge, which is in line with existing research highlighting the importance of somatosensory recovery for achieving good motor recovery.^3,4,11,55^

Further, BBT score at baseline correlated with change in proprioception, which means that some level of active hand use was related to improvement in proprioception. In healthy subjects it has been shown that motor learning stimulates not only motor brain regions but can also lead to changes in sensory function.^57^ In case of stroke patients, the process of motor learning through neurorehabilitation might only be activated for those individuals who have some capability to use the hand. That might be linked to the “virtuous” cycle of activity-dependent plasticity.^58,59^ It is possible that individuals with higher BBT scores at baseline, through spontaneous arm use and general upper limb training throughout the 4 weeks in between assessments, naturally stimulated proprioception, which through a positive feedback loop led to its improvement. With that in mind, it would be beneficial to consider applying proprioception-focused therapies especially for individuals with poor motor function, who may not receive that natural stimulation through active hand use. In fact, proprioceptive facilitation approaches have already been proposed for treatment of hand paresis.^60^ Moreover, proprioceptive training has been shown to result in significant improvements not only in proprioception, but also in motor function, thus it has the potential to enable re-entering the “virtuous” cycle for the severely affected individuals.^61^

### Limitations

From a methodological perspective, the group of considerable improvement in motor function was made based on 3 robotic assessment measures (FF, AROM, EV), while the group of considerable improvement in proprioception was created based on 1 metric (AE), which might have caused a slight imbalance between the groups. Participants were included in the study at different times since stroke (33.89 ± 15.00 days post-stroke), which reflected the actual time when patients arrived in the rehabilitation clinic. This could have influenced the results, since there was a significant difference in time since stroke between the group that considerably improved in either motor function, proprioception or both and the group that changed in neither despite improvement capacity (ie, impaired at baseline in at least 1 modality). However, both groups were less than 3 months since stroke (30.52 ± 12.73 days for the group with considerable improvement and 43.47 ± 16.60 days for the group with no considerable improvement), when spontaneous recovery is still expected to occur. Moreover, in the linear mixed effect model, time since stroke was found to not be an influencing factor neither for the change in proprioception (∆AE), nor for the change in motor function (∆FF, ∆ROM, ∆EV). Lastly, robotic assessments used in this study only evaluated the index finger and it remains to be addressed whether our results generalize to more proximal joints. Nevertheless, correlations of the FF and AE with the assessment of functional hand use suggest that the index finger may indeed be representative as a model for overall hand function.^62^

## Conclusions

A large variability in patterns of changes in hand proprioception and motor function was observed in sub-acute stroke participants. For those where the changes were dissociated, the underlying reason was either that 1 modality was well-preserved at baseline or that sensory/motor tract was affected (absent SSEP/MEP). Among the individuals presenting measurable impairment at baseline, dissociated changes in proprioception and motor function remained frequent (25%). Moreover, functional hand use at baseline was found to be related to improvement in proprioception, while both proprioception and motor function were related to better functional hand use at discharge, indicating an intertwined relationship between proprioception and motor function. Hence, it would be important to monitor those functions regularly to provide more personalized therapies.

## Supplemental Material

sj-pdf-1-nnr-10.1177_15459683231207355 – Supplemental material for The Evolution of Hand Proprioceptive and Motor Impairments in the Sub-Acute Phase After Stroke Supplemental material, sj-pdf-1-nnr-10.1177_15459683231207355 for The Evolution of Hand Proprioceptive and Motor Impairments in the Sub-Acute Phase After Stroke by Monika Zbytniewska-Mégret, Christian Salzmann, Christoph M. Kanzler, Thomas Hassa, Roger Gassert, Olivier Lambercy and Joachim Liepert in Neurorehabilitation and Neural Repair

## Notes

a.
https://www.drks.de/drks_web/navigate.do?navigationId=trial.HTML&TRIAL_ID=DRKS00022952
