# Supplementary material for: The Evolution of Hand Proprioceptive and Motor Impairments in the Sub-Acute Phase After Stroke
Source: Neurorehabil Neural Repair. 2023 Nov 13;37(11-12):823–36. doi: 10.1177/15459683231207355 (PMC10685702; doi:10.1177/15459683231207355)
Supplement: sj-pdf-1-nnr-10.1177_15459683231207355 – Supplemental material for The Evolution of Hand Proprioceptive and Motor Impairments in the Sub-Acute Phase After Stroke [file sj-pdf-1-nnr-10.1177_15459683231207355.pdf]

The evolution of hand proprioceptive and motor impairments in the sub-acute phase after stroke

Monika Zbytniewska-Mégret, Christian Salzmann, Christoph M. Kanzler, Thomas Hassa, Roger Gassert, Olivier Lambercy, Joachim Liepert

12th of September 2023

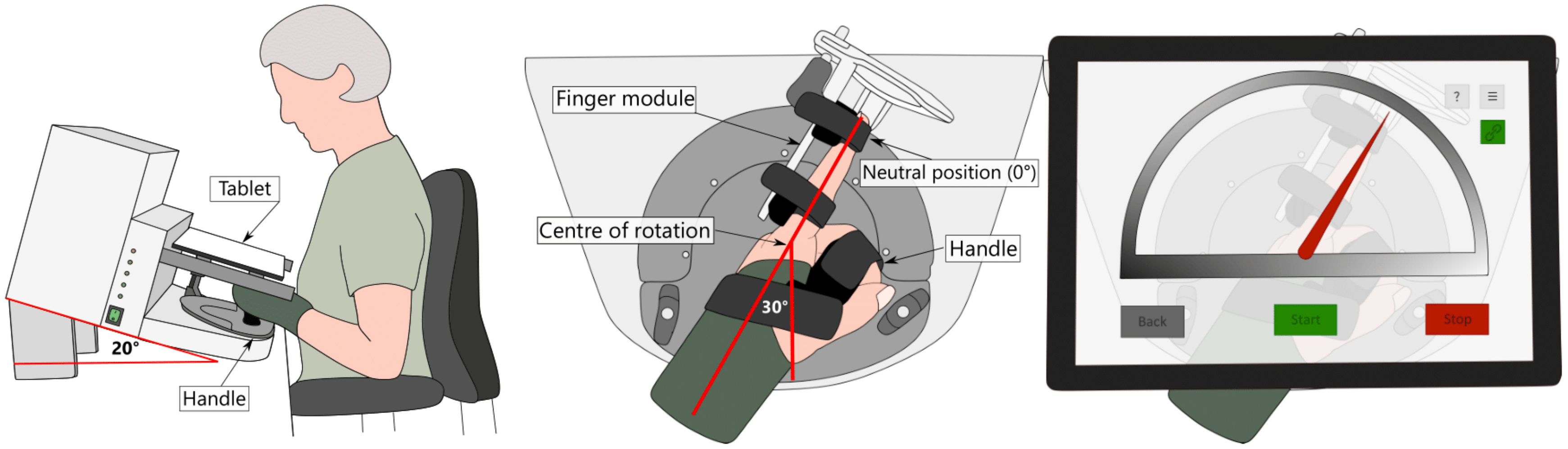

(a) ETH MIKE robot (b) Finger interface (c) Tablet PC

Figure SM1: Schematic of the ETH MIKE robotic platform used for the assessment of index finger metacarpophelangeal joint. (a) Subjects are seated in front of the ETH MIKE robot, with their elbow supported on an arm rest. A wrist splint is worn to avoid any compensatory movements at the wrist. The device is inclined by 20° to minimize parallax errors. (b) The hand is wrapped around a handle, which is set up at the wrist neutral position (0° wrist flexion, 30° from the middle of the device's workspace), ensuring a comfortable resting position for the wrist. The index finger is attached to an adjustable finger module by Velcro straps. The centre of rotation of the end-effector is aligned with the MCP joint. (c) A tablet computer is placed above the hand, removing visual cues from the tested hand and providing an interactive graphical user interface displaying a simple gauge with a red indicator.

Table SM1: The threshold values of robotic and clinical measures used to determine if a participant improved considerably according to a given measure. The values are based on our previous study validating the robotic metrics and for clinical measures on literature (references provided in brackets, full citations are given in the main manuscript). FF: Flexion Force, AROM: Active Range of Motion, EV: Extension Velocity, kUDT: kinaesthetic Up-Down Test, FMA: Fugl-Meyer Upper Limb Motor Assessment, BBT: Box & Block Test, MoCA: Montreal Cognitive Assessment, SRD: Smallest Real Difference (for robotic measures), MDC: Minimal Detectable Change (for clinical measures), Impairment Threshold: defined as age-matched control mean + 2x standard deviation for robotic and taken from literature for clinical measures.

**Measure**	**SRD / MDC**	**Impairment threshold**	**Reference**
AE (°)	9.12	10.63	Zbytniewska et al. 2021
FF (N)	4.88	10.93	
AROM (°)	15.58	63.20	
EV (°/s)	60.68	255.60	
kUDT [0-3]	1	3	Lincoln et al. 1998
FMA [0-66]	5.2	60	Wagner et al. 2008, Meyer et al. 2016
BBT (#/s)	5.5	NaN	Chen et al. 2009
MoCA [0-30]	4	26	Feeney et al. 2016, Nasreddine et al. 2005

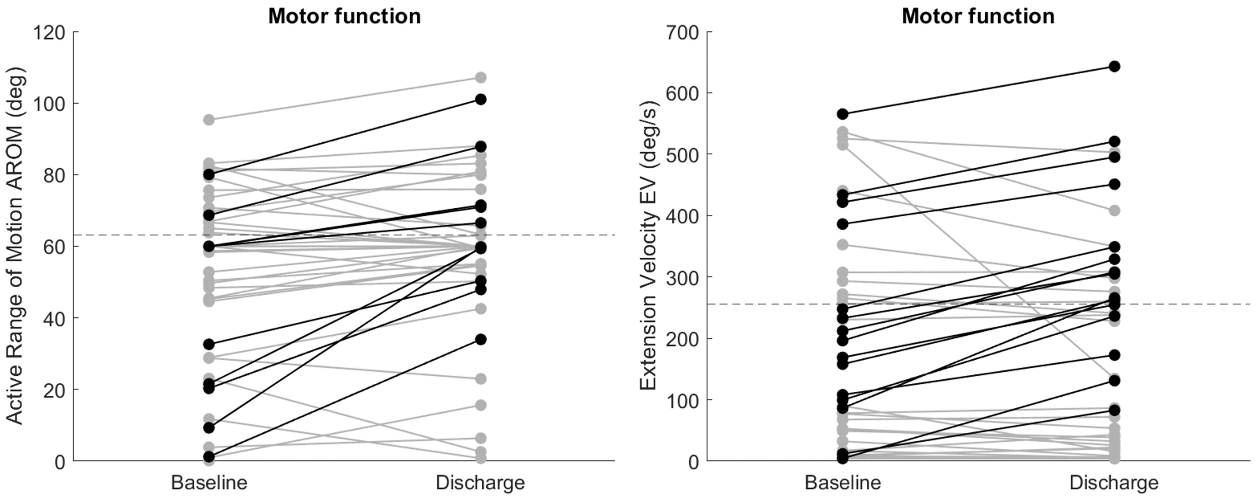

Figure SM2: Changes over time of motor function, measured by two of the robotic task metrics (Active Range of Motion (AROM) and Extension Velocity (EV), two of 3 motor metrics). In black are marked individuals that improved considerably = change larger than the smallest real difference (SRD) or change from impaired to non-impaired. The dashed lines mark impairment thresholds (based on mean + 2SD of neurologically intact age-matched controls). There is a large variability in individual changes. For each metric there are 10, respectively 15, participants that considerably improved (black), but for the majority of the participants changes were too small to be classified as considerable (grey). Higher result indicates better performance.

```
Linear mixed-effects model fit by ML

Model information:
    Number of observations              45
    Fixed effects coefficients           4
    Random effects coefficients         45
    Covariance parameters                2

Formula:
    y ~ 1 + DForce + DROM + DVel + (1 | Subject)

Model fit statistics:
    AIC       BIC       LogLikelihood    Deviance
    276.37    287.21    -132.19          264.37

Fixed effects coefficients (95% CIs):
    Name                   Estimate     SE            tStat      DF    pValue     Lower         Upper
    '(Intercept)'             1.3776      0.78601      1.7527    41    0.08713      -0.20975       2.965
    'DForce'                 0.13855      0.14348     0.96563    41    0.33989      -0.15121     0.42831
    'DROM'                 -0.054333     0.052008     -1.0447    41    0.30228      -0.15937    0.050699
    'DVel'                 0.0096907    0.0079613      1.2172    41    0.23048    -0.0063875    0.025769
```

Figure SM3: Results of the linear mixed effect model - change in proprioception as a dependent variable, change in the subcomponents of motor function, as measured by the robotic assessments, as fixed effects (N=45).

```
Linear mixed-effects model fit by ML

Model information:
    Number of observations              28
    Fixed effects coefficients           4
    Random effects coefficients         28
    Covariance parameters                2

Formula:
    y ~ 1 + DForce + DROM + DVel + (1 | Subject)

Model fit statistics:
    AIC       BIC       LogLikelihood    Deviance
    170.88    178.87    -79.441          158.88

Fixed effects coefficients (95% CIs):
    Name                   Estimate     SE            tStat      DF    pValue      Lower          Upper
    '(Intercept)'             2.3638      0.93547      2.5269    24     0.01851         0.4331      4.2946
    'DForce'                0.091107      0.17867     0.50992    24     0.61476       -0.27765     0.45986
    'DROM'                 -0.068195     0.061156     -1.1151    24     0.27585       -0.19442    0.058025
    'DVel'                   0.01657    0.0081362      2.0366    24    0.052865    -0.00022195    0.033363
```

Figure SM4: Results of the linear mixed effect model – delta proprioception (DProp) as the dependent variable, subgroup analysis (N=28).

```
lme_pm =


Linear mixed-effects model fit by ML

Model information:
    Number of observations             45
    Fixed effects coefficients          9
    Random effects coefficients        45
    Covariance parameters               2

Formula:
    DProp ~ 1 + kUDT + FMA + TSS + Age + Gender + MOCA + StrokeType + Lateralization + (1 | Subject)

Model fit statistics:
    AIC       BIC       LogLikelihood     Deviance
    273.28    293.15    -125.64           251.28

Fixed effects coefficients (95% CIs):
    Name                          Estimate     SE          tStat        DF     pValue       Lower        Upper
    '(Intercept)'                   3.8115       6.3479     0.60043     36      0.55198      -9.0626      16.686
    'kUDT'                          1.2036      0.60568      1.9872     36     0.054545    -0.024745       2.432
    'FMA'                          0.04176     0.029244       1.428     36      0.16192     -0.01755     0.10107
    'TSS'                          0.03751     0.045248       0.829     36      0.41257    -0.054257     0.12928
    'Age'                         -0.10025     0.059491     -1.6852     36       0.1006     -0.22091    0.020399
    'Gender_1'                    -0.68175       1.3927    -0.48952     36      0.62744      -3.5063      2.1428
    'MOCA'                        0.041452      0.11849     0.34983     36       0.7285     -0.19886     0.28177
    'StrokeType_2'                 -2.4328       1.4797     -1.6441     36      0.10887      -5.4338     0.56828
    'Lateralization_1'            -0.64969       1.3171    -0.49328     36      0.62481      -3.3209      2.0215
```

Figure SM5: Results of the linear mixed effect model – change in proprioception, measured by the robotic assessment, as the dependent variable, demographic and stroke-related factors as fixed effects. Abbreviations: kUDT - kinesthetic Up-Down Test, FMA - Fugl-Meyer Assessments, TSS - Time Since Stroke (days), MOCA - Montreal Cognitive Assessment.

Table SM2: Comparison of clinical and robotic measures of proprioception in cases when the considerable improvement groups did not match. In 8 out of 9 cases when improvement in proprioception was detected by the robotic measure, it was in the ceiling of kUDT (kinaesthetic Up-Down). The reason cases when the change according to clinical measure was not detected by robotic were variable, including the change being just below the threshold of considerable improvement (e.g., participant numebr 10 and 12), or it could have also been linked to the subjectivity of the clinical scale. AE: Absolute Error.

#	kUDT T1	kUDT T2	AE (°) T1	AE (°) T2	Improvement detected by
1	3	3	17.74	10.63	Robotic
2	3	3	13.76	9.93	Robotic
3	3	3	13.63	3.84	Robotic
4	3	3	13.60	9.08	Robotic
5	3	3	11.62	8.74	Robotic
6	3	3	10.89	4.82	Robotic
7	3	3	20.85	11.19	Robotic
8	2	2	15.44	9.50	Robotic
9	3	3	18.32	8.77	Robotic
10	0	2	19.52	11.05	Clinical
11	0	1	15.72	22.05	Clinical
12	2	3	14.40	10.82	Clinical
13	1	2	15.32	19.85	Clinical
14	1	2	24.99	24.26	Clinical
15	0	2	9.38	13.19	Clinical
16	2	3	6.67	7.47	Clinical
17	1	2	6.76	20.58	Clinical

```
lme_ff =


Linear mixed-effects model fit by ML

Model information:
    Number of observations             45
    Fixed effects coefficients          9
    Random effects coefficients        45
    Covariance parameters               2

Formula:
    DForce ~ 1 + kUDT + FMA + TSS + Age + Gender + MOCA + StrokeType + Lateralization + (1 | Subject)

Model fit statistics:
    AIC       BIC       LogLikelihood    Deviance
    290.96    310.83    -134.48          268.96

Fixed effects coefficients (95% CIs):
    Name                        Estimate     SE          tStat       DF    pValue     Lower        Upper
    '(Intercept)'                 0.73801      7.7256    0.095528    36    0.92443       -14.93      16.406
    'kUDT'                       -0.70109     0.73714    -0.95109    36     0.3479      -2.1961      0.7939
    'FMA'                        0.022195    0.035592     0.62359    36    0.53683    -0.049988    0.094378
    'TSS'                        0.030238    0.055068     0.54909    36    0.58633    -0.081446     0.14192
    'Age'                       -0.014129    0.072403    -0.19514    36    0.84638     -0.16097     0.13271
    'Gender_1'                     2.0246       1.695      1.1945    36    0.24011       -1.413      5.4621
    'MOCA'                       0.026415     0.14421     0.18317    36    0.85569     -0.26605     0.31888
    'StrokeType_2'                -1.0858      1.8009    -0.60291    36    0.55035      -4.7382      2.5666
    'Lateralization_1'             1.0454      1.6029     0.65216    36    0.51844      -2.2055      4.2963
```

Figure SM6: Results of the linear mixed effect model – change in the Maximum Fingertip Force as the dependent variable, demographic and stroke-related factors as fixed effects. Abbreviations: kUDT - kinesthetic Up-Down Test, FMA - Fugl-Meyer Assessments, TSS - Time Since Stroke (days), MOCA - Montreal Cognitive Assessment.

Table SM3: Comparison of clinical and robotic measures of motor function in cases when the considerable improvement groups did not match. FMA/H: Fugl-Meyer Upper Limb Assessment /Hand subscale, FF: Flexion Force, AROM: Active Range of Motion, EV: Extension Velocity, Improv. detected: considerable improvement detected.

#	FMA T1	FMA T2	FMAH T1	FMAH T1	FF (N) T1	FF (N) T2	AROM (°) T1	AROM (°) T2	EV (°/s) T1	EV (°/s) T2	Improv. detected
1	61	65	14	14	14.32	28.85	68.69	87.82	433.76	520.71	Robotic
2	57	60	14	14	22.80	17.54	82.51	63.24	196.30	328.91	Robotic
3	58	58	14	14	38.45	36.46	80.05	101.02	525.32	502.98	Robotic
4	61	62	14	14	31.26	38.02	73.60	85.37	440.40	349.54	Robotic
5	4	7	0	4	1.22	2.34	20.32	47.94	4.17	4.78	Robotic
6	53	54	14	14	14.50	12.28	59.99	70.92	247.74	348.89	Robotic
7	48	44	14	14	15.48	20.57	60.01	60.00	108.09	172.80	Robotic
8	66	66	14	14	38.84	34.34	95.30	107.10	565.24	642.91	Robotic
9	63	64	14	14	16.80	20.38	63.78	60.01	386.20	451.04	Robotic
10	56	63	10	14	11.20	15.55	75.66	75.91	293.28	276.16	Clinical
11	4	10	0	2	3.07	5.99	45.43	60.00	8.02	22.35	Clinical
12	10	21	0	0	5.49	7.63	48.40	50.22	9.69	20.72	Clinical
13	9	19	1	3	4.18	7.41	50.41	55.10	17.12	43.06	Clinical
14	18	24	4	7	5.48	9.03	44.59	54.59	78.10	86.55	Clinical
15	0	7	0	0	1.46	0.72	23.09	2.60	7.94	6.78	Clinical
16	23	35	5	5	3.15	3.98	58.66	60.00	67.73	71.98	Clinical
17	37	49	11	12	49.55	41.00	59.95	59.97	230.36	238.14	Clinical
18	59	65	12	14	28.25	31.85	70.75	65.70	265.52	227.90	Clinical

```
lme_arom =


Linear mixed-effects model fit by ML

Model information:
    Number of observations              45
    Fixed effects coefficients           9
    Random effects coefficients         45
    Covariance parameters                2

Formula:
    DROM ~ 1 + kUDT + FMA + TSS + Age + Gender + MOCA + StrokeType + Lateralization + (1 | Subject)

Model fit statistics:
    AIC       BIC       LogLikelihood    Deviance
    379.17    399.04    -178.58          357.17

Fixed effects coefficients (95% CIs):
```

Name	Estimate	SE	tStat	DF	pValue	Lower	Upper
'(Intercept)'	14.402	20.587	0.69959	36	0.48868	-27.35	56.154
'kUDT'	-2.795	1.9643	-1.4229	36	0.16337	-6.7788	1.1888
'FMA'	-0.054238	0.094843	-0.57187	36	0.57096	-0.24659	0.13811
'TSS'	-0.18524	0.14674	-1.2624	36	0.21493	-0.48286	0.11237
'Age'	-0.02378	0.19294	-0.12325	36	0.90259	-0.41507	0.36751
'Gender_1'	1.9206	4.5167	0.42523	36	0.6732	-7.2396	11.081
'MOCA'	0.33765	0.38428	0.87865	36	0.38542	-0.44171	1.117
'StrokeType_2'	-2.8162	4.7989	-0.58683	36	0.56098	-12.549	6.9165
'Lateralization_1'	-1.7106	4.2715	-0.40047	36	0.69118	-10.374	6.9523

Figure SM7: Results of the linear mixed effect model – change in Active Range of Motion (AROM) as the dependent variable, demographic and stroke-related factors as fixed effects. Abbreviations: kUDT - kinesthetic Up-Down Test, FMA - Fugl-Meyer Assessments, TSS - Time Since Stroke (days), MOCA - Montreal Cognitive Assessment.

```
lme_vel =


Linear mixed-effects model fit by ML

Model information:
    Number of observations             45
    Fixed effects coefficients          9
    Random effects coefficients        45
    Covariance parameters               2

Formula:
    DVel ~ 1 + kUDT + FMA + TSS + Age + Gender + MOCA + StrokeType + Lateralization + (1 | Subject)

Model fit statistics:
    AIC      BIC       LogLikelihood    Deviance
    540.7    560.57    -259.35          518.7

Fixed effects coefficients (95% CIs):
    Name                        Estimate     SE         tStat        DF    pValue      Lower       Upper
    '(Intercept)'                 117.65      123.9      0.94956     36     0.34867     -133.63     368.94
    'kUDT'                       -4.5642     11.822     -0.38607     36     0.70171      -28.54     19.412
    'FMA'                        0.80499    0.57081       1.4103     36     0.16705    -0.35267     1.9626
    'TSS'                       0.056562    0.88318     0.064043     36     0.94929     -1.7346     1.8477
    'Age'                        -2.0156     1.1612      -1.7358     36    0.091145     -4.3706    0.33936
    'Gender_1'                   -48.454     27.183      -1.7825     36    0.083109     -103.58     6.6771
    'MOCA'                        2.6916     2.3128       1.1638     36     0.25216     -1.9989     7.3822
    'StrokeType_2'               -1.0176     28.882    -0.035232     36     0.97209     -59.594     57.559
    'Lateralization_1'           -25.629     25.708     -0.99695     36     0.32544     -77.767     26.508
```

Figure SM8: Results of the linear mixed effect model – change in the Maximum Velocity Extension as the dependent variable, demographic and stroke-related factors as fixed effects. Abbreviations: kUDT - kinesthetic Up-Down Test, FMA - Fugl-Meyer Assessments, TSS - Time Since Stroke (days), MOCA - Montreal Cognitive Assessment.

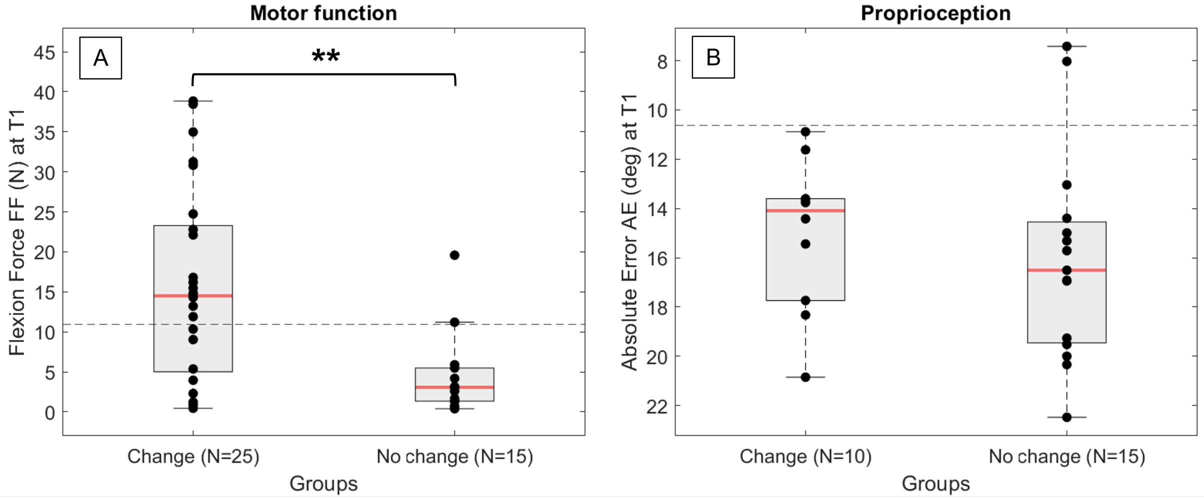

Δ Motor function group	FF T1 (N)	AROM T1 (°)	EV (°/s)
Change (N=25)	15.83 ± 12.03	56.26 ± 24.32	221.74 ± 184.74
No change (N=15)	4.46 ± 5.05	41.52 ± 25.28	80.36 ± 140.53
KW p-val.	**0.003**	**0.009**	**0.026**

Δ Proprioception group	AE T1 (°)
Change (N=10)	15.03 ± 3.11
No change (N=15)	16.06 ± 4.27
KW p-val.	0.3181

Figure SM9: (A) The difference in baseline motor function in the group that did and did not considerably improve in motor function is significant for all motor metrics (FF, AROM, EV). The group "no change" means no change in neither proprioception nor motor function and only considering individuals impaired at baseline. (B) The difference in baseline proprioception (measured by AE) is not significant between the groups that did and did not improve in proprioception.

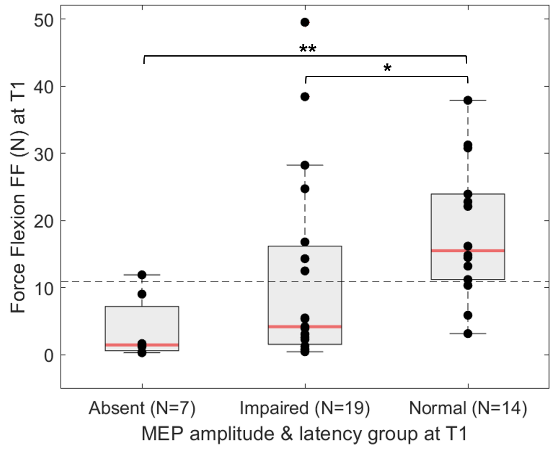

(a) Flexion Force at T1 vs MEP at T1

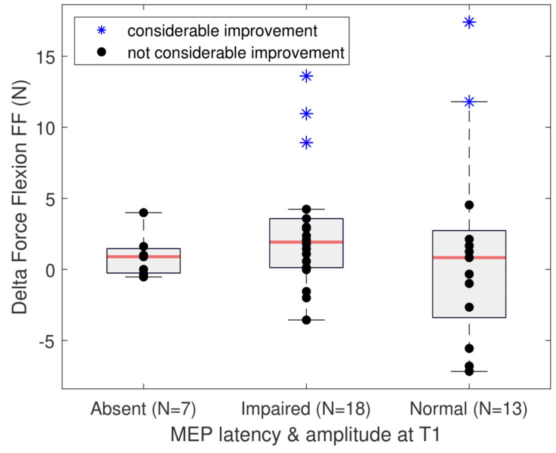

(b) Change in Flexion Force vs MEP at T1

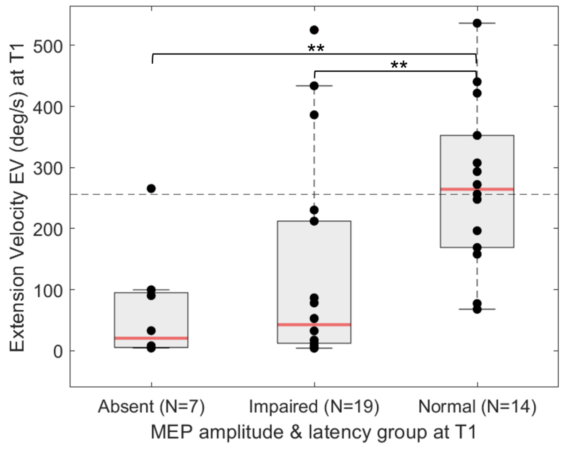

(c) Active Range of Motion at T1 vs MEP at T1

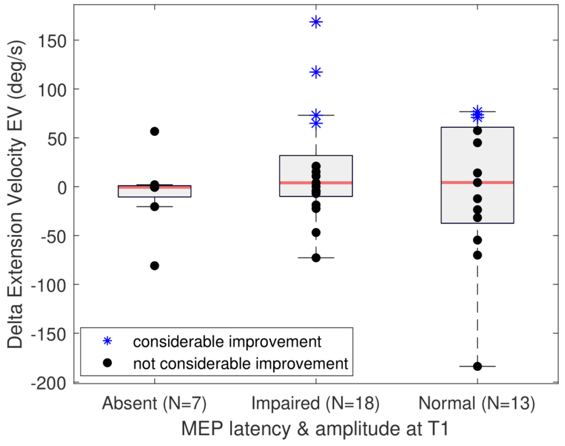

(d) Change in Active Range of Motion vs MEP at T1

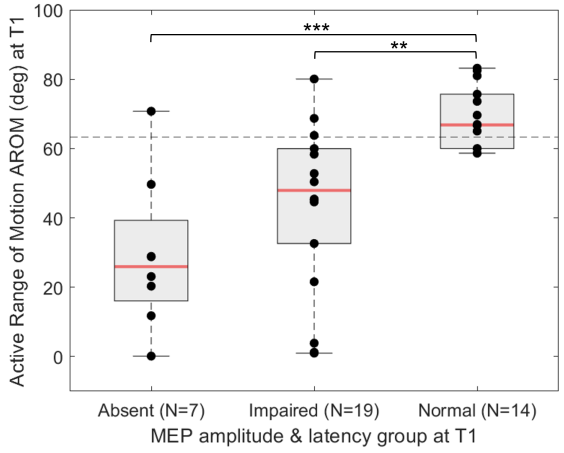

(e) Extension Velocity at T1 vs MEP at T1

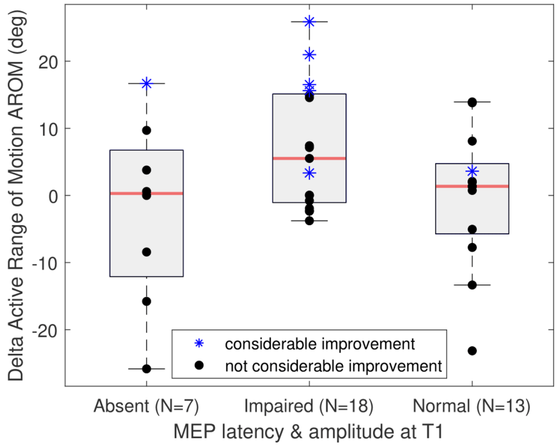

(f) Change in Extension Velocity vs MEP at T1

Figure SM10: Grouping of participants based on MEP scores to compare motor metrics FF, AROM and EV at T1 and delta FF, AROM and EV between the absent, impaired, and normal MEP response groups. In left panels the dashed line indicates impairment threshold. Larger values indicate better performance and more improvement. The reason for "Impaired" and "Normal" groups having missing data for right panel is due to the missing discharge measurement for these patients (no delta FF, AROM, EV available).

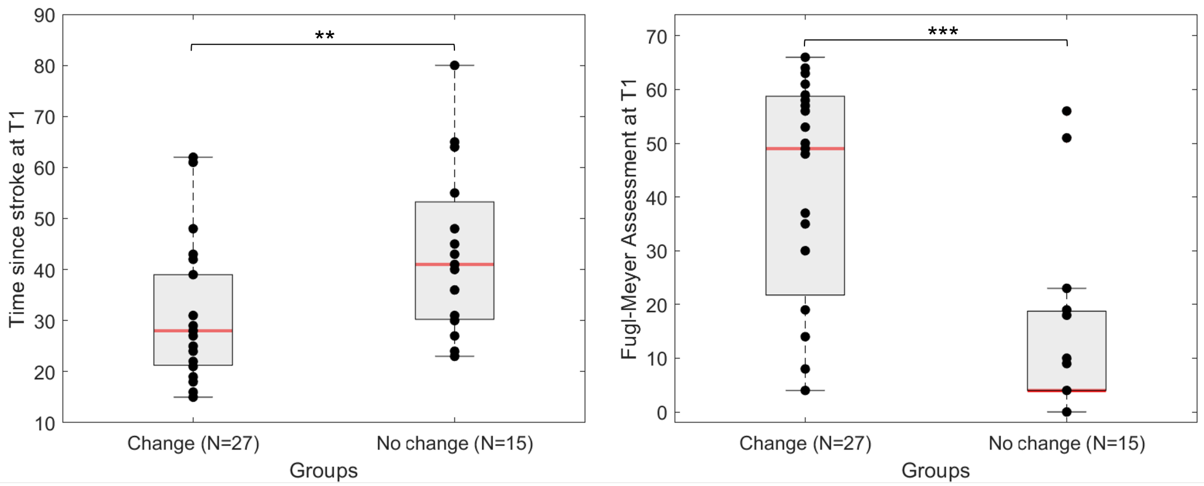

Group	Time Since Stroke (days)	Age (years)	Gender	Type of stroke	Lateralization (LHS/RHS)	Cognition at T1 (MoCA)
Change (N=27)	30.52 ± 12.73	66.59 ± 10.41	67% Males	19% haemorrhagic	52% RHS	21.78 ± 4.34
No change (N=15)	43.47 ± 16.60	67.93 ± 10.63	53% Males	33% haemorrhagic	67% RHS	21.60 ± 7.06
KW p-val.	**0.008**	0.703	0.400	0.286	0.358	0.377

Figure SM11: The difference between the group that did not change and the group that changed in at least one function (motor or proprioception) in terms of demographic and stroke-related factors – time since stroke, age at baseline, gender, type of stroke (haemorrhagic or ischemic), lateralization (right or left hemispheric stroke) and cognitive function (measured by Montreal Cognitive Assessment). Individuals that were not impaired in neither proprioception nor motor function at baseline (N=3) were not included in this analysis.

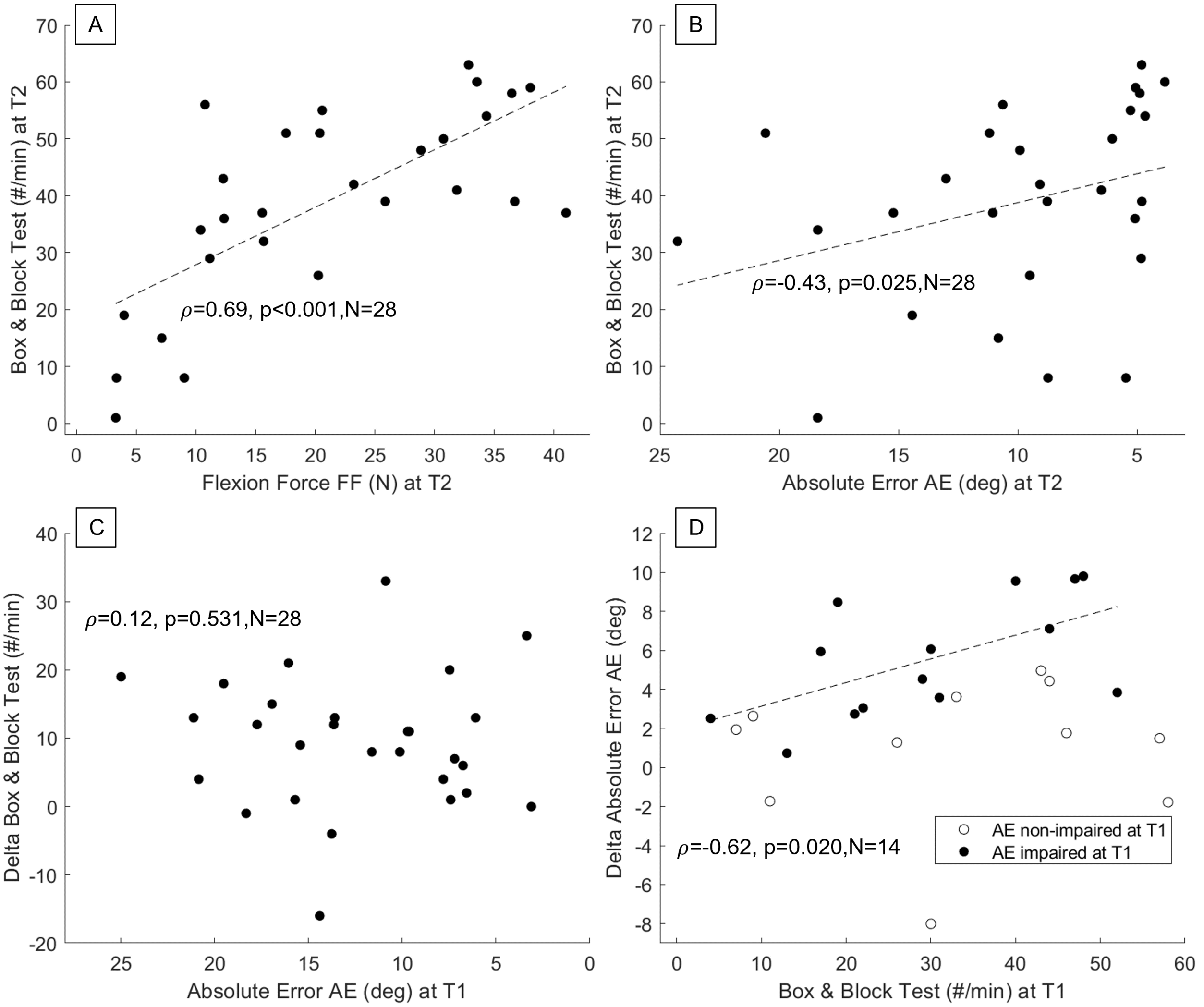

Figure SM12: The relationship between hand impairments and functional hand use when all values in the floor effect of the Box & Block Test (BBT) were removed. The results remained the same. Both motor function (here Force Flexion, A) and proprioception (Absolute Error, B) correlated with the skilled hand use at discharge, measured by Box & Block Test (BBT). C) Impaired proprioception at inclusion did not correlate with functional recovery, as measured by delta BBT. D) At least partially preserved hand function was needed for improvement in proprioception, especially considering individuals with improvement capacity (i.e., impaired at T1).

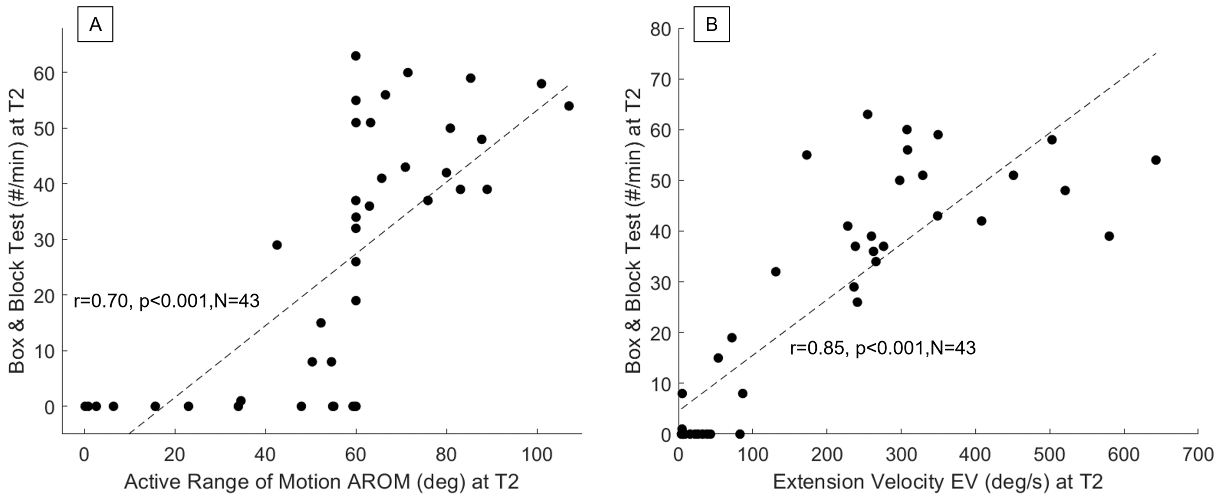

Figure SM13: The correlation between motor hand impairments and functional hand use at discharge was strong for both Active Range of Motion (AROM) and Extension Velocity (EV).

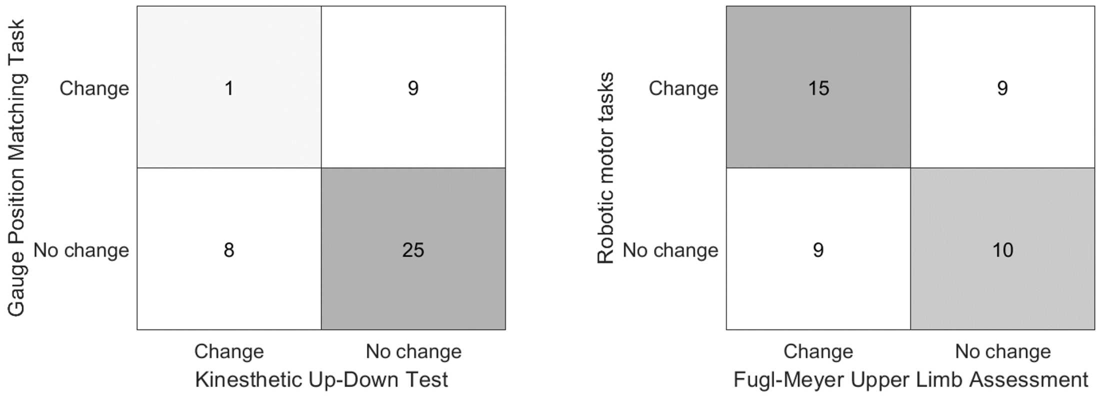

Figure SM14: Classification agreement between clinical and robotic measures (60% agreement between proprioception measures, 58% agreement between motor measures). Change here means considerable improvement, defined for robotic measures as change larger than the smallest real difference (SRD) or change from impaired to non-impaired. For kUDT considerable change was selected as change by 1 point, and for FMA it was equal to the Minimal Detectable Change (MDC), 5.2 points or improved above 60 points. The characteristics of the group that changed in robotic task but not in clinical in proprioception are that these subjects were in the ceiling of the clinical scale. In motor tasks it was possible to detect subtle changes for individuals severely affected using robotics. Changes not detected by robotics but captured in FMA can be explained by the fact that this scale considers the whole upper limb, not only the hand.

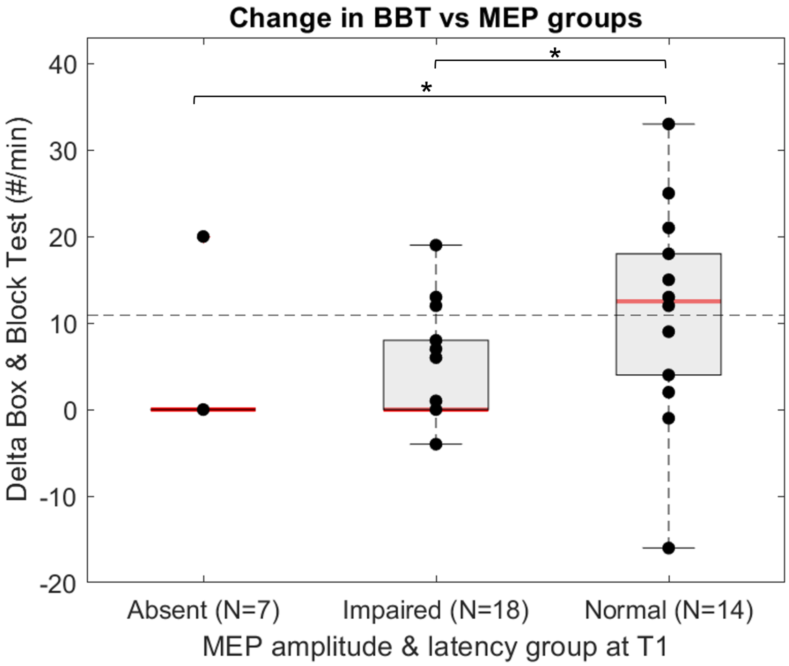

Figure SM15: Grouping of participants based on MEP scores to compare the change in Box & Block Test (BBT). Participants with normal MEP response showed biggest improvement in BBT.

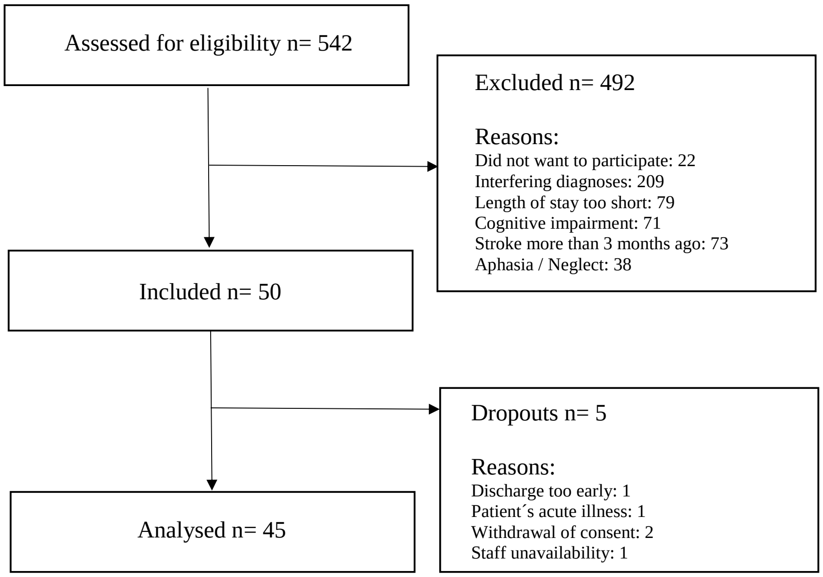

Figure SM16: PRISMA diagram representing the number of patients screened, included and analysed for the study, together with the exact reasons for exclusion.
